# Comparison of DNA extraction methods on different sample matrices within the same terrestrial ecosystem

**DOI:** 10.1038/s41598-024-59086-4

**Published:** 2024-04-15

**Authors:** Giulio Galla, Nadine Praeg, Theresa Rzehak, Else Sprecher, Filippo Colla, Julia Seeber, Paul Illmer, Heidi C. Hauffe

**Affiliations:** 1 Conservation Genomics Research Unit, Research and Innovation Centre, Fondazione Edmund Mach, S. Michele all’Adige, Italy; 2 Department of Microbiology, Universität Innsbruck, Innsbruck, Austria; 3 Institute for Alpine Environment, EURAC Research, Bolzano, Italy; 4 Department of Ecology, Universität Innsbruck, Innsbruck, Austria; 5 National Biodiversity Future Center (NBFC), S.c.a.r.l., Palermo, Italy

**Keywords:** 16S rRNA gene, Amplicon-sequencing, Metataxonomy, Method evaluation, MICCA, Microbial ecology, Microbiome

## Abstract

Metataxonomic studies of ecosystem microbiotas require the simultaneous processing of samples with contrasting physical and biochemical traits. However, there are no published studies of comparisons of different DNA extraction kits to characterize the microbiotas of the main components of terrestrial ecosystems. Here, and to our knowledge for the first time, five DNA extraction kits were used to investigate the composition and diversity of the microbiota of a subset of samples typically studied in terrestrial ecosystems such as bulk soil, rhizosphere soil, invertebrate taxa and mammalian feces. DNA extraction kit was associated with changes in the relative abundance of hundreds of ASVs, in the same samples, resulting in significant differences in alpha and beta diversity estimates of their microbiotas. Importantly, the impact of DNA extraction kit on sample diversity varies according to sample type, with mammalian feces and soil samples showing the most and least consistent diversity estimates across DNA extraction kits, respectively. We show that the MACHEREY–NAGEL NucleoSpin® Soil kit was associated with the highest alpha diversity estimates, providing the highest contribution to the overall sample diversity, as indicated by comparisons with computationally assembled reference communities, and is recommended to be used for any large-scale microbiota study of terrestrial ecosystems.

## Introduction

The soil ecosystem is a complex, three-dimensional habitat for many taxa of microorganisms, (bacteria, fungi, archaea and viruses) that provide essential ecosystem services, such as soil fertility, critical to human survival. The transmission of microorganisms from soil to plants and animals, maintains and enhances the microbiotas of these species, which are essential for their development and health^1^. For example, plants secrete organic substances into soils in the form of root exudates, which aid the recruitment of specialized members of soil microbial communities into plant rhizosphere^2^; at the same time, bulk soil is one of the most important contributors to plant endophytic microbiota. Soil dwelling invertebrates such as bacterial-feeding roundworms and earthworms feed on soil microorganisms, but they also influence soil microbial community directly (as in the cases of roundworms) or indirectly, by contributing to soil structure and nutrient availability^3–5^. Furthermore, the deliberate or involuntary consumption of soil (‘geophagy’) contributes commensal microorganisms to the animal gut microbiota; in fact, up to 3% of the rumen microbiome of sheep and cattle can be traced back to the soil^1^. Additionally, according to the biodiversity hypothesis^6,7^, contact with natural environments, as well as ingestion of unprocessed and fermented foods, is expected to enrich human microbiota, promote immune balance, and protect against allergies and inflammatory disorders. Characterizing the compositional similarities and co-occurrences across microbial taxa found in soil, plants and animals (both vertebrates or invertebrates, living below and above the soil) is of pivotal importance for understanding the role of microbiota in the One Health framework, as well in the conservation and management of natural and traditional agricultural ecosystems.

Metataxonomics has recently become the most widely adopted method for studying complex microbial communities, using short read sequencing of amplicons generated by targeting specific hypervariable regions of key conserved genes^8–10^. This analytical strategy has now been applied to the investigation of microbiota in a great variety of terrestrial and aquatic ecosystems on Earth^11–15^ and even in space^16^. However, the application of amplicon sequencing-based metataxonomy approaches to the understanding of microbiota interactions across the soil ecosystem requires the simultaneous profiling of microorganisms from various matrices, such as soil, plant roots, invertebrates, and animal faeces (often used as a proxy for gut microbiota). Purification of DNA extracts suitable for amplicon-sequencing from environmental samples could be hampered by the co-extraction of secondary metabolites with enzymatic inhibitory effects such as humic substances or fulvic acids from soil samples^17^ as well as complex polysaccharides, bile salts, lipids, and urate from fecal samples^18^. Furthermore, the extraction of sufficient amounts of DNA for subsequent metataxonomic analyses could be challenging in the case of microscopic animals such as soil dwelling roundworms or intestinal helminths^19^ due to their small size and limited biomass. Progress in this field has been made possible, at least in part, by the availability of a number of commercial kits allowing the extraction of nucleic acids suitable for PCR amplification from a wide variety of samples, regardless of their physical properties (e.g. size, mass, physical state) and chemical composition (e.g. water content, presence and abundance of PCR inhibitors)^18^. However, each DNA extraction protocol has its own specifications (e.g. buffers composition, lysis conditions), and these methodological choices throughout the workflow of a metataxonomic study can greatly affect the results^20–23^. In fact, DNA extraction has been shown to be the main contributor to the distortion of bacterial abundance from their original values, by altering, for instance, the abundance of taxa more difficult to lyse (e.g. gram-positive bacteria^22,24^). Consequently, in all cases where sample types are processed with different DNA extraction methods, the technical variation resulting from DNA extraction has the potential to bias alpha and beta diversity estimates^24,25^, limiting our ability to compare datasets. As a result, microbial ecology studies focusing on community assemblies in diverse sample types require a workflow minimizing technical variation, starting with the adoption of a DNA extraction kit suitable for the sample types being studied.

Thus far, comparative methodological studies have largely focused on single sample types, such as stool^20,24,26,27^, soil^28–30^ and invertebrates^31–34^. To the best of our knowledge, only a single eDNA-oriented study has been published that compared extraction kits for a range of sample types, including water, litter and soil^35^. However, this study did not include many sample types that are of pivotal importance for understanding microbial co-occurrences (and potential interactions) between plants, soil, invertebrates, and vertebrates.

Here, with the aim of quantifying differences in microbiota composition between the main actors of terrestrial ecosystems, we tested five commercial DNA extraction kits on two soil components, two taxa of soil invertebrates, and two above-ground vertebrate taxa from the same sample site. Standard metataxonomics methods and bioinformatics software were then applied to: (i) investigate the extent to which kits specifically designed for a given sample type can be used for the processing of other sample types; (ii) estimate the extent to which variation in diversity estimates for each sample type or taxon can be associated with DNA extraction method; (iii) define quantitative variation in taxa abundances resulting from DNA extraction methods; (iv) identify the most suitable kit for terrestrial ecosystem microbiota studies.

## Results

### DNA extraction efficiency varies across kits and sample types

Genomic DNA was successfully extracted from all samples of each sample type with all DNA extraction kits tested: DNeasy® Blood & Tissue (QIAGEN; hereafter QBT), QIAamp® DNA Micro (QIAGEN; QMC), NucleoSpin® Soil (MACHEREY–NAGEL; MNS), DNeasy® PowerSoil® Pro (QIAGEN; QPS) and QIAamp® Fast DNA Stool Mini (QIAGEN; QST) (Figure S1). However, we did not find a single kit that outperformed all others for the entire set of investigated sample types (Figure S1). QST was the best performing kit for Feces_hare samples in terms of DNA concentration, but not for other sample types, including Feces_cattle; in fact, QST produced significantly lower DNA quantity than other kits. Additionally, despite QMC being commercialized for small sample sizes, it was among those providing the highest DNA concentrations for invertebrate (Invert_beetle, Invert_roundworm), bulk soil (Soil_horizonA) and rhizosphere soil (Soil_rhizosphere) samples, but provided yields similar to QBT, MNS and QPS for Feces_hare, although not for Feces_cattle. Considering the 260/280 ratio, we found significant differences in purity of DNA extracts across kits (Figure S2A); with QST providing the highest values. For the 260/230 ratio, MNS provided the best performances regarding the 260/230 ratio in all samples except for Invert_beetle (Figure S2B). Of note, despite the observed variability in DNA concentration and purity, the V4 region of the 16S rRNA gene was successfully amplified using the eluted DNA from all kits and sample types. Sequencing of the resulting 284 libraries (including extraction and PCR negative controls) generated a median number of 46,283 sequence reads per library, ranging from 15,945 (St_NP4_3_mc) to 216,074 (St_SP4_3_mc).

### Differences in the extraction efficiency of gram-positive bacteria

**Figure 1:**
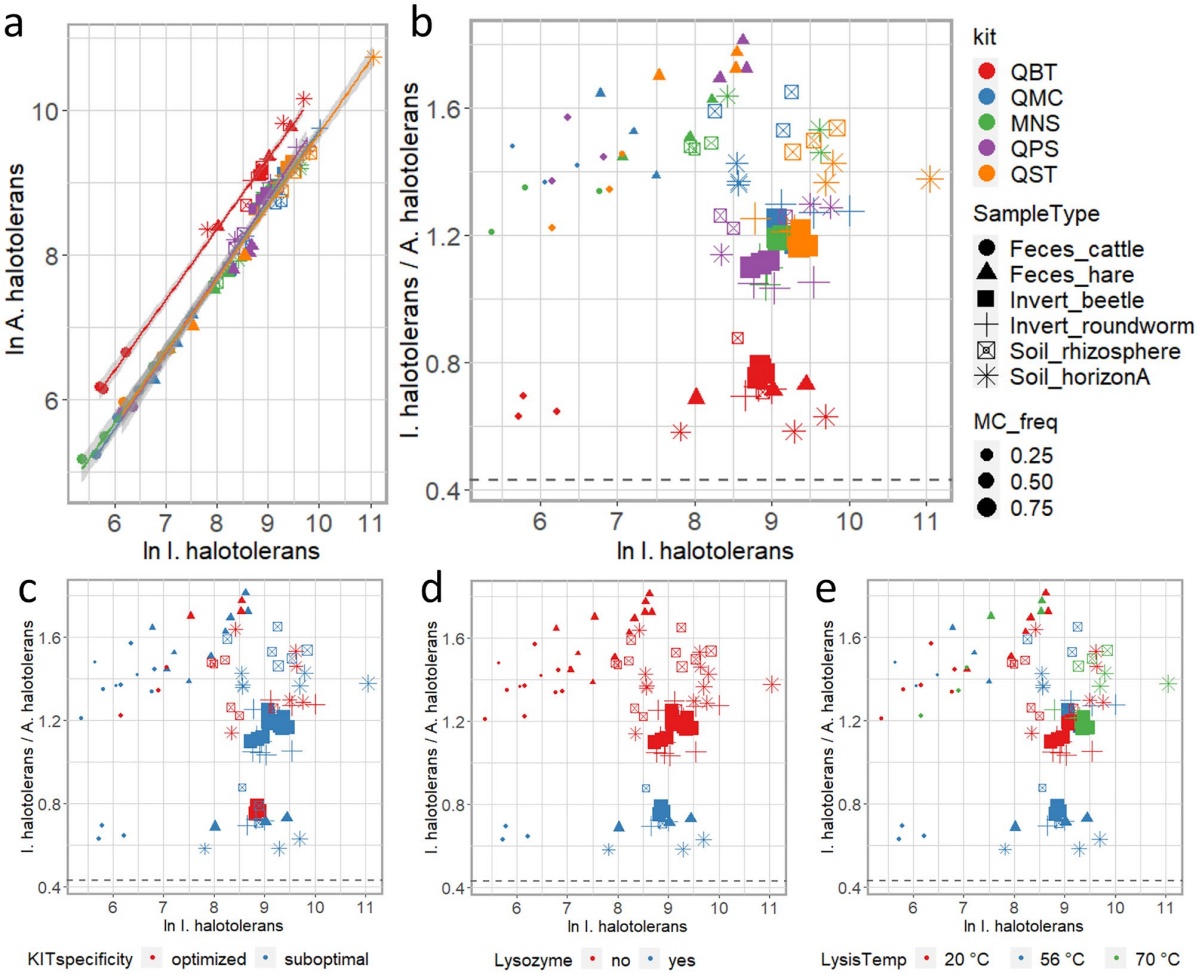
DNA extraction efficiency of the gram-positive bacterium *A. halotolerans.* Panel a: for each sample type and DNA extraction method, the ln *A. halotolerans* sequence counts (y axis) are plotted as a function of ln *I. halotolerans* sequence counts (x axis). For each DNA extraction method (kit) the regression line with 95% confidence interval is shown. Panels b-e: for each sample type and DNA extraction method, the ratios between *A. halotolerans* and *I. halotolerans* sequence counts (y axis) are reported as a function of ln *I. halotolerans* sequence counts (x axis). Point size varies with the percentage of MC-related sequences over the total number of sequences as reported in the legend (MC_freq). Sample types are marked with different shape formats, as indicated in the plot legend (SampleType). (**a**, **b**): Samples are colored according to the DNA extraction method as indicated in the plot legend (kit). (**c**–**e**): Samples are colored according to the specificity of adopted kits (**c**), to inclusion of lysozyme in lysis buffers (**d**) and lysis temperature (**e**). KITspecificity was defined as follows: we considered QBT specific for Invert_beetle; QMC for Invert_roundworm; MNS and QPS specific for both soil samples and QST specific for both fecal samples; all other kit-sample type pairs were considered suboptimal.

To assess the extraction efficiency of gram-positive bacteria we included a commercial mock community (MC) composed by a known number of cells of two halotolerant bacteria *I. halotolerans* (gram-negative) and *A. halotolerans* (gram-positive) with each combination of sample type and kit for aliquots D1mc, D2mc, and D3mc (Fig. 1A and B). The abundance of MC-related ASVs relative to the total number of reads ranged from 1.4% (QMC-BP4-3-mc) to 91.1% (QMC-NP4-3-mc) (Fig. 1A and B). The mean ratio between ASV abundance of the two MC-derived bacteria was 0.71 ± 0.08 for QBT, 1.40 ± 0.15 for QMC, 1.35 ± 0.19 for MNS, 1.31 ± 0.25 for QPS and 1.39 ± 0.19 for QST (Fig. 1B). Although the observed values were higher than the expected 0.43, based on the composition of the MC provided by the supplier, in all combinations of sample type and DNA kit (Fig. 1), QBT always produced the lowest ratio regardless of sample type (mean value: 0.71 ± 0.08), indicating that this kit had the highest extraction efficiency of *A. halotolerans* (Fig. 1A, B) of these five kits. While we found a clear connection between the ratios of the two taxa and the use of lysozyme during DNA extraction (Fig. 1D), we found no association between this ratio and the adoption of a sample-specific kit (Fig. 1C), a particular lysis temperature (Fig. 1E), or homogenization strategy and lysis time (data not shown).

### DNA extraction method affects microbial diversity estimates

Taxonomic classification of assembled ASVs shows that a distinct microbial community characterizes technical and biological replicates of each sample type (Figure S3). Clostridia and Bacteroidia were the most represented Classes found in Feces_cattle. These two classes were highly represented in Feces_hare as well, but the microbial community of these latter samples were also rich in Gammaproteobacteria and unclassified Firmicutes. We found that taxa belonging to the classes Gammaproteobacteria and Bacilli were the most abundant in our samples of Invert_beetle, followed by Clostridia, Bacteroidia and Alphaproteobacteria (Figure S3). The two most represented classes of bacteria in Invert_roundworm were Gammaproteobacteria and Alphaproteobacteria. Soil_horizon A and Soil_rhizosphere also displayed distinct different microbial communities, although the most represented taxa found in both were Alphaproteobacteria, Gammaproteobacteria and Actinobacteria. While Soil_rhizosphere also had a high proportion of Bacteroidia, Soil_horizonA were further enriched in Blastocatellia, Verrucomicrobia, Vicinamibacteria and Thermoleophilia, among others (Figure S3).

**Figure 2:**
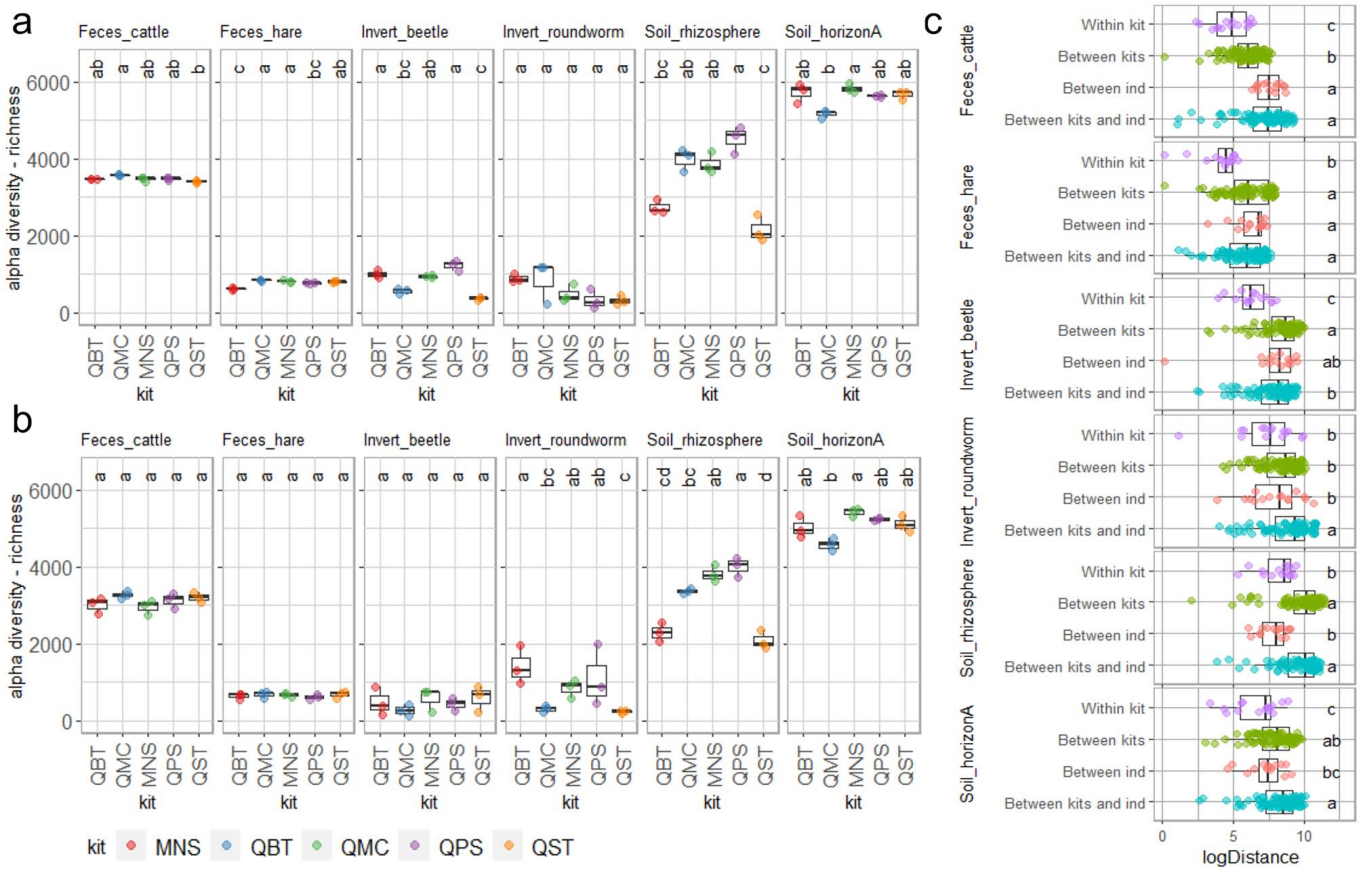
Diversity estimates across sample types and kits. Panel a: richness (S) observed in technical replicates. Panel b: richness (S) observed in biological replicates. Small letters on figure tops in panel a and b indicate significant differences according to DNA extraction kit (Kruskal–Wallis, *p* < 0.05). Samples are clustered by sample type and colored according to the DNA extraction method as indicated in the plot legend (kit). QBT: DNeasy® Blood & Tissue (QIAGEN); QMC: QIAamp® DNA Micro (QIAGEN); MNS: NucleoSpin® Soil (MACHEREY–NAGEL); QPS: DNeasy® PowerSoil® Pro (QIAGEN); QST: QIAamp® Fast DNA Stool Mini (QIAGEN). Panel c: Log2 Euclidean distances between richness (S) estimates across samples and DNA extraction methods. Euclidean distances were estimated within each kit (*Within kit*), between kits (*Between kits*), between individuals (*Between ind*.) as well as between kits and individuals (*Between kits and ind*.). Small letters within the boxes indicate significant differences (Kruskal–Wallis tests, *p* < 0.05).

Alpha diversity estimates for each sample type and DNA extraction kit are shown in Fig. 2 and S4. Both indices considered in this study highlighted significant differences in diversity estimates across DNA extraction methods (Figs. 2, S4, Table S2). Of note, we found no significant association between alpha diversity estimates and purity of DNA extracts expressed as 260/230 ratio (Table S3). Instead, richness estimates in Invert_beetle were negatively associated with the concentration of DNA eluates (Richness: t-value − 2.89; *p*-value: 0.0078; Table S3) and purity of DNA extracts expressed as 260/280 ratio (Richness: t-value − 2.75; *p*-value: 0.0108). At the same time, alpha diversity estimates were positively associated with concentration of DNA eluates in Soil_rhizosphere (Richness: t-value 4.03; *p*-value: 0.0005; Shannon: t-value 3.90; *p*-value: 0.0006; Table S3) and 260/280 ratio and Soil_horizonA (Richness: t-value 2.26; *p*-value: 0.0325; Table S3).

The extraction kits providing significantly higher alpha diversity estimates were QMC for Feces_cattle, QMC and MNS for Feces_hare, QBT and QPS for Invert_beetle, QPS for Soil_rhizosphere and MNS for Soil_horizonA. No significant difference in alpha diversity estimates across kits was detected for Invert_roundworms. While for Feces_cattle, Feces_hare and Invert_beetle, the variability across biological replicates (Fig. 2B) superseded the differences between DNA extraction methods (Fig. 2A), Soil_horizonA and Soil_rhizosphere had significantly different diversity estimates across kits in both technical and biological replicates (Fig. 2A, B). In fact, Euclidean distances of estimated richness (S) were significantly higher in pairwise comparisons *Between kits* than *Within kit* in all sample types, except for Invert_roundworm (Fig. 2C). Instead, we found no significant differences in Euclidean distances of estimated richness for pairwise comparisons *Between kits* and *Between individuals* in most sample types, except for Feces_cattle and Soil_rhizosphere. Interestingly, for Soil_rhizosphere, Euclidean distances of estimated richness were lower in pairwise comparisons *Between sampling sites* (reported as *Between individuals* in the corresponding Figure) than *Between kits* (Fig. 2C).

**Figure 3:**
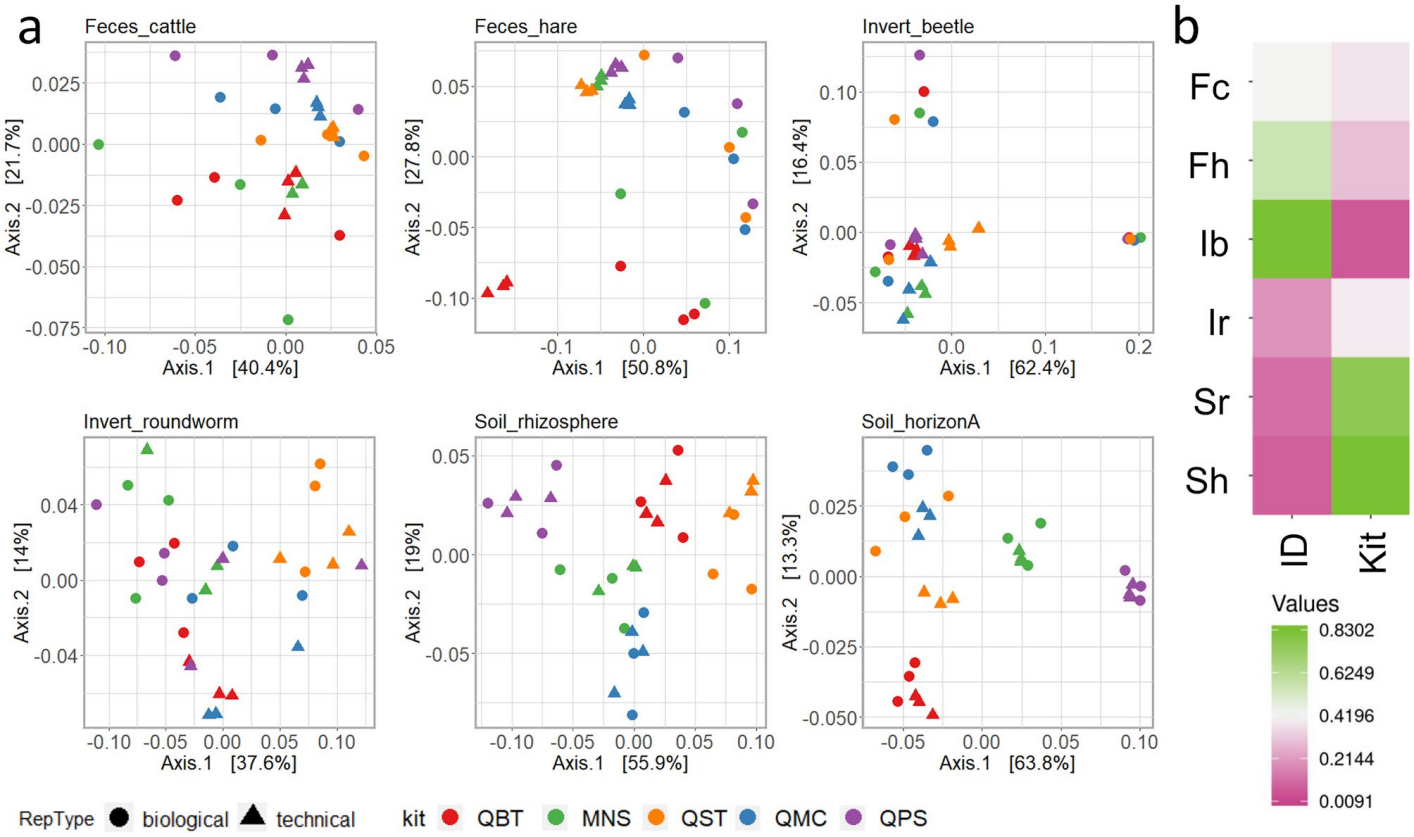
Beta diversity estimates. Panel a: Beta diversity estimates (weighted UniFrac) on samples processed with different DNA extraction protocols. Technical and biological replicates are shown. Each plot refers to a different sample type. Samples are colored according to the DNA extraction method as indicated in the plot legend (kit). Technical and biological replicates are marked with different dot shapes as reported in the figure legend. QBT: DNeasy® Blood & Tissue (QIAGEN); QMC: QIAamp® DNA Micro (QIAGEN); MNS: NucleoSpin® Soil (MACHEREY–NAGEL); QPS: DNeasy® PowerSoil® Pro (QIAGEN); QST: QIAamp® Fast DNA Stool Mini (QIAGEN). Panel b: Variance in diversity estimates explained in PERMANOVA analyses (Values refer to R^2^) by the following variables: individual (ID) and DNA extraction method (kit). Fc: Feces_cattle; Fh: Feces_hare; Ib: Invert_beetle; Ir: Invert_roundworm; Sr: Soil_rhizosphere; Sh: Soil_horizonA.

Between-sample diversity estimates were computed as weighted UniFrac (Fig. 3) and Bray–Curtis (Figure S5) distances. PERMANOVA statistical tests performed on the entire set of samples (N = 180, replicates without MC) to test the impact of DNA extraction method on beta diversity estimates, after controlling for the variation associated with the different type of samples, showed that a significant fraction of variation in these estimates could be explained by DNA extraction methods alone (Unifrac R^2^: 0.03, *p*-value ≤ 0.001; Bray–Curtis R^2^: 0.02, *p*-value ≤ 0.001; Table S4) and by the interaction between sample type and DNA extractions method (Unifrac R^2^: 0.06, *p*-value ≤ 0.001; Bray–Curtis R^2^: 0.07, *p*-value ≤ 0.001; Table S4). At the level of single sample types, PERMANOVA statistical tests on UniFrac diversity estimates showed that a large fraction of variation in these estimates is associated with the extraction kit used (R^2^ ranging from 0.299 to 0.830, *p*-values ranging from 0.010 to 0.001; Fig. 3A and B, Table S4). Furthermore, our data showed that extraction kit is the main driver of diversity estimates in Soil_horizonA (R^2^: 0.830, *p*-value < 0.001, Fig. 3, Table S4), Soil_rhizosphere (R^2^: 0.764, *p*-value < 0.001, Fig. 3, Table S4) and Invert_roundworm (R^2^: 0.389, *p*-value < 0.001, Fig. 3, Table S4). Interestingly, although pool ID is the main driver of diversity in Feces_cattle and Feces_hare (R^2^: 0.427 and 0.555, *p*-values < 0.001, respectively), a fraction of this diversity (e.g. R^2^: 0.427 and 0.555, *p*-values < 0.001, respectively) was associated with DNA extraction methods for these sample types as well (Fig. 3, Table S4).

**Figure 4:**
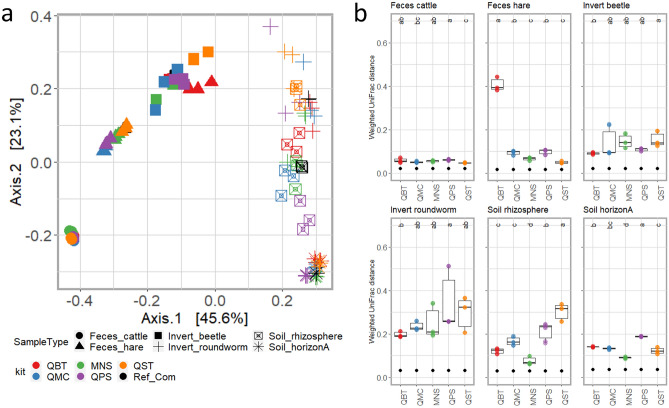
Beta diversity estimates across reference communities and corresponding samples. Panel a: PCoA was generated by using weighted UniFrac distances. The percentage of the total variance explained by the axis is reported in the figure. Sample types are marked with different shape formats as indicated in the plot legends (SampleType). Samples are colored according to the DNA extraction method as indicated in the plot legend (kit). Panel b: Distance across trans-samples (black dots) and the corresponding samples. Samples are colored according to the DNA extraction kits as indicated in the plot legend (kit). Letters in the above area of graphs indicate the grouping resulting from Kruskal–Wallis tests on UniFrac distances between trans-samples and the corresponding samples extracted with different kits.

As an index of closeness of each sample type to the ‘true’ microbial composition, our reference microbial communities that were computationally assembled for each sample type by merging the fastq files from each DNA extraction method, were used to retrospectively measure the contribution of each library to these reference communities (Figs. 4A, S5). The clustering of all samples based on weighted UniFrac (Fig. 4A) and Jaccard (Figure S6) distances placed each reference community in proximity to the samples from which they were generated. In addition, we also found some significant differences in diversity estimates between reference communities and corresponding samples (Figs. 4B, S5), which, together with the observed clustering pattern, further indicates that kits adopted for DNA extraction affect composition (e.g. the presence of a given taxa, Figs. 4, S6) and frequency (Fig. 4) of identified taxa. Referring to the UniFrac diversity estimates, kits with the lowest distances between samples and corresponding and reference communities, and hence providing the highest contribution to their assemblage, were QST and QMC for Feces_cattle, QST and MNS for Feces_hare and MNS for soil (Soil_horizonA and Soil_rhizosphere) (Fig. 4B). In the case of invertebrates (Invert_beetle, Invert_roundworm), we found QBT and most of the kits (including MNS) providing similar contributions to the assembly of corresponding reference communities. In fact, as shown in Fig. 4B, for all sample types except Soil_horizonA and Soil_rhizosphere, we found several DNA extraction kits performing similarly in terms of distances between corresponding and reference communities and providing estimates comparable to that between reference communities and their closest kit (e.g. “QMC” for *Bos*, “MNS” for *Lepus*). Inspection of each library's contribution to the corresponding reference communities using Jaccard distance estimates, which differently from weighted UniFrac only account for presence-absence data, further highlighted the significantly better performance of MNS for soil samples (Soil_horizonA, Soil_rhizosphere), invertebrates (Invert_beetle, Invert_roundworm) and Feces_hare (Figure S6).

### Significant changes in ASV abundance due to DNA extraction methods

**Figure 5:**
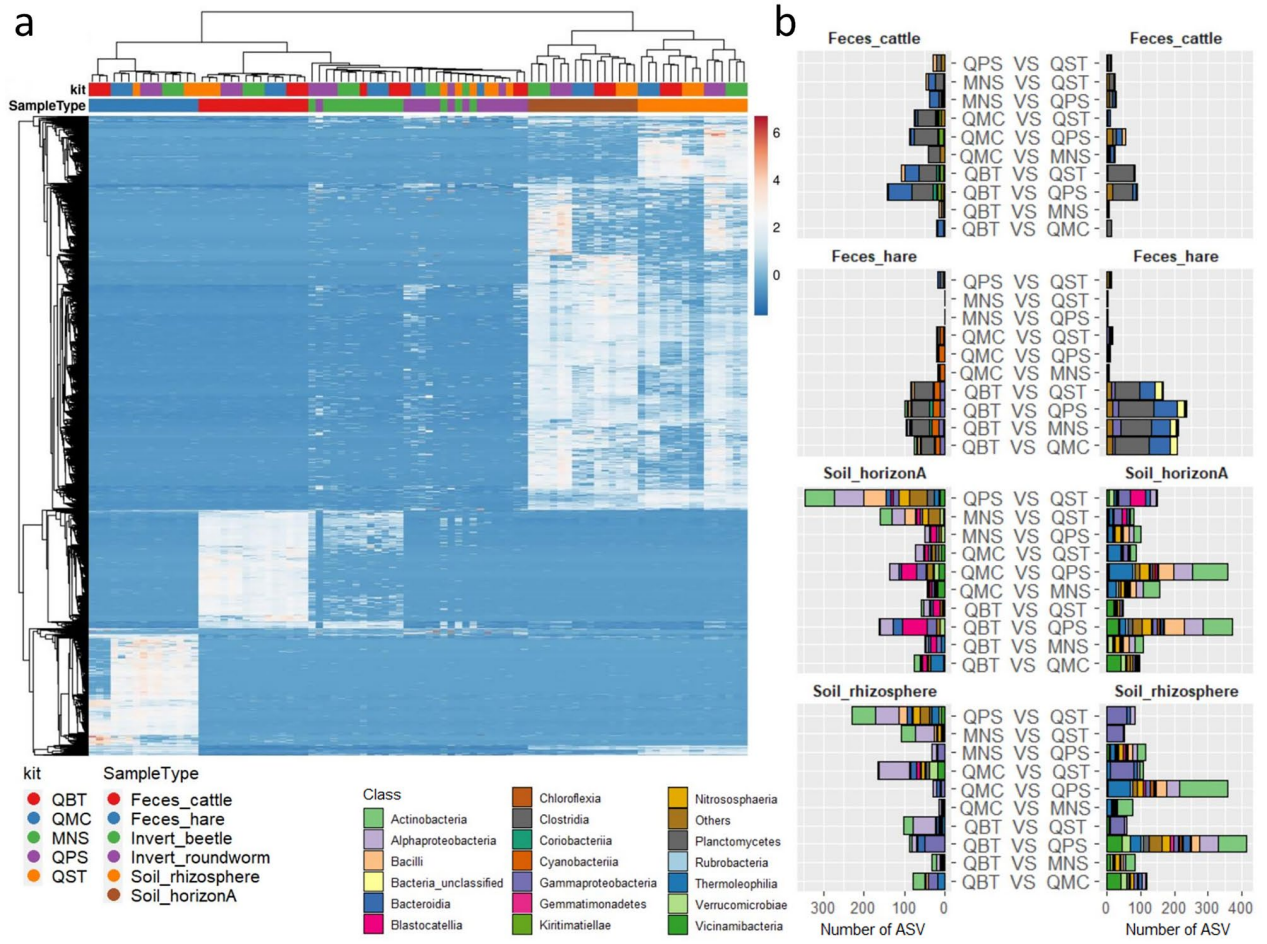
Clustering of libraries based on differential ASVs and taxonomic classification of differential ASVs. Panel a: Clustering of libraries (N: 90) based on the abundance of ASVs displaying significant differences in at least one comparison across kits (ASV N: 2105) as identified with DeSeq2. Original values were ln(x + 1)-transformed. Rows (ASVs) were centered; unit variance scaling was applied to rows. Rows and Columns (libraries) were clustered using Manhattan distance and average linkage. The heatmap has 2105 rows and 90 columns. Samples (SampleType) and DNA extraction methods (kit) are colored according to the legend (bottom left). Panel b: Taxonomic classification of ASVs with significant differences in their abundance across DNA extraction methods. Bar plots were generated by using ASVs with significant differences in their abundance across kits as identified with DESeq2. ASV counts were agglomerated at the taxonomic level of Class. For each pairwise comparison between kits, the left bar shows the taxonomic classification of ASVs more abundant in the first kit, while the right bars show the taxonomic classification of ASVs more abundant in the second kit. Classes are colored according to the reported legend (bottom right). QBT: DNeasy® Blood & Tissue (QIAGEN); QMC: QIAamp® DNA Micro (QIAGEN); MNS: NucleoSpin® Soil (MACHEREY–NAGEL); QPS: DNeasy® PowerSoil® Pro (QIAGEN); QST: QIAamp® Fast DNA Stool Mini (QIAGEN).

Taxa significantly enriched or depleted by the adopted DNA extraction kit were identified by pairwise comparisons across kits in all sample types except for Invert_beetle (Fig. 5A, Table S5). The number of differential ASVs across kits varied considerably (Table S5). Soil_horizonA and Soil_rhizosphere displayed the highest variation across kits, with an observed number of differential ASVs ranging from about 100 (for the contrasts: QMC vs MNS and QBT vs QST, respectively) to more than 500 (for the contrast QBT vs QPS in both cases). For Feces_cattle and Feces_hare the number of ASVs with significant differences in pairwise comparisons ranged from less than 10 (for the Feces_hare contrasts: MNS vs QST and MNS vs QPS; Table S5) to 334 (for the contrast QBT vs QPS in Feces_hare; Table S5). The clustering of libraries based on the abundance of differential ASVs (Fig. 5A) highlighted again a clear separation between soil and fecal microbial communities and, within each sample type, further emphasized the separation of libraries generated from DNA extracts of different kits (Fig. 5A). Of note, and in agreement with our beta diversity estimates, both soil samples (Soil_horizonA and Soil_rhizosphere) were associated with a clear separation between kits specifically designed for soil (e.g. MNS and QPS) and the other kits (e.g. QBT, QMC, QST) (Fig. 5A).

When differential ASVs were collapsed to the taxonomic level of class, we found several bacterial and archaeal taxa whose abundance was significantly enriched and/or depleted by the DNA extraction method (Fig. 5B, Tables S5, S6). Bacterial classes affected in fecal samples (Feces_cattle and Feces_hare) were mainly Clostridia, Bacteroidia, Bacteria_unclassified and Cyanobacteria. This latter Class was significantly enriched in Feces_hare (but not Feces_cattle) only when extracted with QMC and QBT. Likewise, the adoption of alternative DNA extraction methods significantly modulated the relative abundance of Actinobacteria, Alphaproteobacteria and Gammaproteobacteria in Soil_horizonA and Soil_rhizosphere, Blastocatellia in Soil_horizonA as well as Thermoleophilia, Vicinamibacteria and Nitrososphaeria in Soil_rhizosphere. Soil_horizonA and Soil_rhizosphere extracted with the QPS were associated with a depletion in Blastocatellia, Gammaproteobacteria (along with Bacteroidia and Verrucomicrobiae) and an enrichment in Actinobacteria, Bacilli, Nitrososphaeria and Thermoleophilia for all pairwise comparisons, except when MNS was included in the comparison (Fig. 5, Tables S5, S6).

## Discussion

The microbial communities of terrestrial ecosystems represent study areas of great interest for microbial ecology with potential applications to biodiversity conservation^36,37^. Research projects focusing on complex ecosystems frequently require the simultaneous processing of biological matrices with non-uniform physical and bio-chemical properties, for which the adoption of methodological strategies minimizing technical variability across project samples is of pivotal importance. The present study deepens our understanding of the impact of DNA extraction methods on diversity indices of soil, soil invertebrates and vertebrate fecal sample bacterial microbiota (Table 1). We show that DNA extraction methods provide DNA eluates with different quantitative and qualitative profiles. Although all tested DNAs were successfully amplified and sequenced, we found that kit selection can bias both alpha and beta diversity estimates of resulting microbial communities. Additionally, switching DNA extraction kit is reflected in significant differences in the abundance of hundreds of ASVs. Interestingly, however, the impact of DNA extraction kit on estimated sample diversity varied according to the sample type, with some type of samples, such as Feces_cattle, showing highly consistent results across kits and other samples, such as Soil_horizonA and Soil_rhizosphere, displaying marked and reproducible signs of kit-associated bias.

**Table1:** Summary of diversity estimates and differential abundance testing.

Sample type (abbreviation)	Kits with the highest α diversity (S) estimates	Pattern of variation (S)^a^	Main β diversity driver	Kit with highest contribution to the reference community^b^	Number of DATs (max–min)	DATs taxonomy (Class)
Feces cattle (Feces_cattle)	All, except QST	W kit < B kit** < **B ind	Individual	QST, QMC	231–22	*Clostridia, Bacteroidia*
Feces hares (Feces_hare)	QMC, MNS, QST	W kit < B kit **≈** B ind	Individual	QST, MNS	334–3	*Clostridia, Bacteroidia, Cyanobacteriia*
Beetles (Invert_beetle)	QBT, QPS, MNS	W kit < B kit **≈** B ind	Individual	All, except QST	nd	nd
Roundworms (Invert_roundworm)	All	W kit **≈** B kit **≈** B ind	Kit	All, except QPS	16 – 1	*Alphaproteobacteria, Thermoleophilia, Bacteroidia, Bacilli*
Rhizosphere Soil (Soil_rhizosphere)	QPS, QMC, MNS	W kit < B kit > B ind	Kit	MNS	501 – 90	*Actinobacteria*, *Alphaproteobacteria, Gammaproteobacteria, Thermoleophilia, Vicinamibacteria, Nitrososphaeria*
HorizonA soil (Soil_horizonA)	All, except QMC	W kit < B kit **≈** B ind	Kit	MNS	537 – 108	*Actinobacteria*, *Alphaproteobacteria, Gammaproteobacteria, Blastocatellia*

For each sample type, the DNA extraction kit resulting in the highest α diversity estimates (S), the comparison of patterns of variation in S between extraction kits and replicates, the main driver of β diversity, and the kit with the highest contribution to the assembly of the corresponding reference communities are reported. The maximum and minimum number of taxa with significant differences in their abundance in all pairwise comparisons (DATs) across the five DNA extraction kits, and the main taxonomic Classes associated with these DATs are also reported. QBT: DNeasy® Blood & Tissue (QIAGEN); QMC: QIAamp® DNA Micro (QIAGEN); MNS: NucleoSpin® Soil (MACHEREY–NAGEL); QPS: DNeasy® PowerSoil® Pro (QIAGEN); QST: QIAamp® Fast DNA Stool Mini (QIAGEN). nd: not detected.

^a^W kit: within kit; B kit: between kits; B ind.: between individual samples. ^b^Referred to UniFrac distance estimates.

Direct comparisons across DNA extraction methods were initially made using quantity and quality of DNA extracts from the wide range of combinations between sample types and DNA extraction methods considered in this study. The performance of each kit varied between and within sample types. We did not find a single kit clearly outperforming the others in terms of concentration of DNA extracts in all sample types (Figure S1). As we focused on the amplification of a short target region (16S-V4: ~ 250 bp excluding primer binding sites) from both environmental and non-invasive samples, which are notoriously prone to high DNA degradation^38^, we considered purity (e.g. 260/280 and 260/230 ratios from UV/VIS spectra) rather than DNA integrity^24^ as key factor for direct assessment of DNA extraction efficiency. Three kits among those tested (namely, MNS, QPS, QST) make use of specific strategies for the removal of secondary inhibitors, such as inhibitor removal columns (MNS), or inhibitor removal buffers (namely IRT and InhibitEX Buffer for QPS and QST, respectively). We found that DNA extracted with MNS were characterized by the highest purity values for the 260/230 ratio across all samples except for Invert_beetle. Higher effectiveness of MNS compared to QPS, QST, and PowerFecal kit from Mo Bio Laboratories Inc. (which was not tested in this study) in extracting high purity DNA from raw feces and lagoon effluents was also reported by^39^. Although a previous study on fecal samples collected from different animal species failed to find a clear correlation between the DNA purity and success of subsequent amplification and sequencing^40^, efficient removal of potential inhibitors of enzymatic activity is considered of pivotal importance for metataxonomics, as high contaminant concentration may lead to reduced PCR amplification, and consequently, to skewed composition of microbial communities^24,40–42^. 

The inclusion of a commercial mock community (MC) with each combination of sample type and kit (Fig. 1) provided the opportunity to infer the extraction efficiency of gram-positive bacteria across different commercial kits. The mean ratio between ASV abundance of the two MC-derived bacteria, which based on the MC composition and 16S rRNA gene copy number in the two MC taxa is expected to be ~ 0.43, varied across DNA extraction methods, with QBT displaying higher performances in the extraction of *A. halotolerans* irrespectively of the sample type and abundance of MC-related ASVs detected in each library. Interestingly, since the extractions with the QBT kit were performed by making an optional pretreatment for gram-positive bacteria that uses lysozyme to promote cell wall lysis of these microorganisms, our data support the idea that lysozyme might be an effective alternative to other widely adopted strategies for cell lysis such as bead beating (adopted by MNS and QPS), prolonged lysis time (as in the case of overnight lysis for QMC) and/or high lysis temperatures (as in the case of QST, in which lysis is performed at 70 °C). Although the fine tuning of MC dose for the different types of sample (and associated microbial communities) was not needed to efficiently estimate the extraction efficiency of gram-positive bacteria, our data pinpoint the importance of calibrating the MC dose in different sample types to avoid overpowering the sample bacterial community (as seen in Invert_roundworm samples), as this would lead to a markedly distorted representation of community data^43^.

We found compelling evidence that composition and diversity estimate of sample microbial communities are affected by DNA extraction kit (Figs. 2, 3, 4, 5). However, the increased efficiency in the extraction of the gram-positive bacteria *A. halotolerans* observed for QBT did not translate into higher alpha diversity estimates, a finding that could be explained assuming that *A. halotolerans* is not a good proxy for the extraction efficiency of gram-positive bacteria. Unfortunately, as previously highlighted by^43^, since its recent release only a few studies have used this product, and information regarding its correct use and potential practical limitations in non-model sample types is still scarce. Alternatively, we might consider that gram-positive bacteria only represent a small fraction of considered communities. However, despite the strong discrepancies between estimates made with different methodologies^44^, several studies report that gram-positive bacteria account for a significant fraction of fecal^44–46^ and soil^47,48^ microbiota. In fact, kit providing significantly higher alpha diversity estimates across the different sample types considered in our study were QMC for Feces_cattle, QMC and MNS for Feces_hare, QBT and QPS for Invert_beetle, QPS for Soil_rhizosphere and MNS for Soil_horizonA (Table 1, Fig. 2). Importantly, alpha diversity estimates indicated that differences associated with DNA extraction methods can be of the same order of magnitude than differences detected across biological replicates (Fig. 2). Moreover, regardless of the amplitude of estimated diversity, variation in richness (S) across technical replicates processed with the different kits (*Between kits;* Table 1) was generally comparable or higher than variation detected between biological replicates of the same sample type (*Between individuals*), and both were higher than variation estimated across technical replicates processed with the same method (*Within kit*) in all investigated samples except Invert_roundworms. Despite that variation in beta diversity estimates across all samples was driven by the sample types (Unifrac, permanova R^2^: 0.779, *p*-value < 0.001; Bray–Curtis, permanova R^2^: 0.631, *p*-value < 0.001), a smaller but significant portion of variation in diversity estimates was associated with the kit alone (Unifrac, permanova R^2^: 0.027, *p*-value < 0.001; Bray–Curtis, permanova R^2^: 0.021, *p*-value < 0.001) and its interaction with sample type (Unifrac, permanova R^2^: 0.059, *p*-value < 0.001; Bray–Curtis, permanova R^2^: 0.070, *p*-value  < 0.001). Soil samples showed the greatest difference in alpha diversity across kits, and consistently, kit was the main driver of beta diversity estimates in Soil_horizonA (Unifrac, permanova R^2^: 0.830, *p*-value < 0.001), Soil*_* rhizosphere (Unifrac, permanova R^2^: 0.764, *p*-value < 0.001) and soil-dwelling animals (Invert_roundworm; Unifrac, permanova R2: 0.389, *p*-value < 0.001) (Fig. 3, Table S4). However, a significant fraction of diversity was associated with the kit also in our vertebrate fecal samples: Feces_hare (Unifrac, permanova R^2^: 0.298, *p*-value < 0.01) and Feces_cattle (Unifrac, permanova R^2^: 0.378, *p*-value < 0.001). Our findings for soil are in agreement with a previous study highlighting that variation in diversity estimates due to DNA extraction method in soil might overwhelm biological differences due to contrasting land uses^41^, and confirm those from a previous investigation^23^, but are in contrast with those reported by^30^, who found no differences in diversity estimates associated with three DNA extraction methods (none of which were tested in our study). Regarding fecal samples, previous benchmarking studies on human fecal samples^24^ have shown the importance of mechanical lysis and bead beating for the efficient extraction of DNA from gram-positive bacteria and highlighted the good performance of MNS in the extraction of fungal taxa for metagenomic studies^49^.

Although the reference communities adopted in this study can not provide an exhaustive representation of original communities, as they are (by definition) dependent on the subset of samples adopted for their assembly, their use as reference communities allowed us to rank the diverse kits based on their distance (expressed as weighted UniFrac or Jaccard dissimilarity) from the corresponding reference communities, thus providing a valuable indication of the effectiveness of each method to embrace the complexity of each sample and its microbial community. Consistent with the presented diversity estimates, the contribution of each DNA extraction method to the assembly of the reference communities was, again, highly dependent on sample type (Figs. 2, S5). Thus, while technical replicates of invertebrate animals (Invert_beetle and Invert_roundworm) were prone to compositional differences when processed with alternative DNA extraction methods (Fig. 4 and S6), other sample types such as soil (Soil_horizonA and Soil_rhizosphere) as well as Feces_hare were prone to variation in both composition and frequency between observed taxa (as indicated by weighted UniFrac distances; Fig. 4). According to the weighted UniFrac distance, the kit providing the lowest distance between samples and corresponding reference communities were the following: QST and QMC for Feces_cattle, QST and MNS for Feces_hare and MNS for both types of soil samples (e.g. Soil_horizonA and Soil_rhizosphere). Conversely, invertebrate samples were characterized by several kits (including QBT and MNS) providing similar UniFrac distance estimates with the corresponding reference communities. Consistently, switching DNA extraction kit resulted in the identification of hundreds of ASVs with significant differences in their abundance across kits^50^. This was observed in all sample types, except Invert_beetle, for which the lack of significant differences across methods could reflect the observed high variability in diversity estimates which was observed across replicates. Two clear examples of variation associated with the DNA kit are, for example, the enrichment of Cyanobacteria in Feces_hare extracted with QBT and QMC as well as the enrichment of Actinobacteria, Alphaproteobacteria, Bacilli and Nitrososphaeria observed in Soil_horizonA and Soil_rhizosphere extracted with QPS and MNS. The enrichment of gram-positive bacteria (e.g. Actinobacteria, Bacilli) and other bacterial classes difficult to lyse such as Alphaproteobacteria^51^ in both soil samples extracted with QPS and MNS was consistent with earlier observations that mechanical lysis was the most efficient method for the extraction of DNA from gram-positive and spore-forming bacteria^24,28,52,53^. The observation that an increase in gram-positive bacteria was mirrored by an apparent depletion of taxa belonging to Gammaproteobacteria, Bacteroidia and Gemmatimonadetes is also consistent with previous studies^24,28,49,54^ and can be explained by competition between DNA fragments/amplicons in compositional datasets^55^. Interestingly, the list of taxa modulated by DNA extraction includes microorganisms involved in fundamental ecosystem processes such as phosphorus cycling (Vicinamibacteria^56^) as well as nitrification and methane oxidizers (Nitrososphaeria^57–59^), which emphasizes that different extraction methods might affect the outcomes and conclusions of ecological studies, and why uniformity of extraction methods is desirable.

Despite the constant decrease of amplicon-sequencing costs, resources limitations most frequently imply that only a limited number of highly referenced DNA extraction methods and types of samples could be compared before any study. This study addresses the impact of DNA extraction methods on a wide range of sample types by testing highly referenced DNA extraction methods selected among the many available. Comparisons between selected methods demonstrate the impact of switching DNA extraction kit varies across sample types, with some sample types, such as bovine feces, being resilient to technical variation and others, such as bulk soil, rhizosphere soil and invert roundworms, being particularly vulnerable to technical variation derived from DNA extraction methods. Therefore, the selection of the kit to be used in projects involving different sample types (such as feces and soil or invertebrate animals) should be done by considering the project specific sample susceptibility to methodological variation (see Table 1) and by extracting the DNA from most resilient samples (such as mammalian feces) using methods specifically designed for the set of samples showing higher sensitivity to DNA-derived technical variation (such as soil samples). Using different DNA extraction kits leads to significant differences in the abundance of several bacterial classes and reproducible changes in alpha and beta diversity estimates and thus actively contributes to shape the sample microbial community as addressed by conventional metataxonomic pipelines. From the comparison of the tested kits, we noted that QST was generally associated with reduced alpha diversity estimates and poor contribution to the theoretical reference community, in particularly for soil, rhizosphere and invertebrate animals (Invert_beetle). QBT, QMC and QPS were among kits providing the highest alpha diversity estimates in mammalian feces, invertebrate animals, rhizosphere and bulk soil samples. However, these kits typically performed worse than MNS regarding the relative contribution to the theoretical reference community, even in invertebrate and soil samples. Conversely, eluates generated with MNS were associated with the highest alpha diversity estimates (in all sample types except Invert_beetle) and greater contribution to the corresponding theoretical microbial communities (in all sample types except Feces_cattle). These observations indicate that MNS is very effective in capturing the composition and diversity of most samples considered in this study and for this reason, we recommend its use for any large-scale ecological study on microbial communities, especially those studies comparing soil and rhizosphere with other fundamental actors of terrestrial ecosystems.

Future microbial ecology studies involving the simultaneous processing of different sample types, such as soil, feces and invertebrate animals would benefit from higher methodological harmonization as this would facilitate comparisons of amplicon sequencing data in complex ecological systems. Similarly, greater methodological harmonization would facilitate the study of the functional potential present or expressed by microbial communities found in these heterogeneous contexts through the application of metagenomics and metatranscriptomics, leading to a better understanding of the contribution of the microbiota to the adaptive potential and health of animals, plants, and soil to changing environments, which is of pivotal interest to One Health studies.

## Methods

### Study area

All samples were collected from dry grassland pastures at the Long-Term (Socio-) Ecological Research (LT(S)ER) area in Val Mazia/Matschertal (Vinschgau Valley, South Tyrol, Italy; site code LTER_EU_IT_097, 46.6928°, 10.6157°; https://deims.org/11696de6-0ab9-4c94-a06b-7ce40f56c964) in June-July 2019. The LT(S)ER in Val Mazia/Matschertal is managed by the Institute for Alpine Environment (EURAC) and comprises different land-uses and various forest types with an elevational ranging from 950 to 3700 m a.sl.

### Above-ground vertebrates

Fresh fecal samples from Pezzata Rossa Italiana cattle (hereafter Feces_cattle) were collected from two sites at 2000 m a.s.l. Each of nine samples was collected from a single freshly deposited cowpat using sterile tweezers (three collection points per pat) and placed in sterile 50 ml polypropylene tubes. A total of nine single fresh hare (*Lepus* spp.) fecal pellets (Feces_hare) at least 20 m apart were collected using sterile tweezers from five sites located between 1000 and 2000 m a.s.l. and placed singly in sterile 15 ml polypropylene tubes. All fecal samples were stored at − 20 °C up to 24 h before being transferred to the laboratories of Fondazione E. Mach where they were archived at − 80 °C until pooling and DNA extraction. Separately for each of the two species, three to five fecal samples (Table S1) were pooled to generate technical and biological replicates as follows: for each pool approximately 0.5 g of frozen feces from each sample were placed in a sterile pestle and mortar and ground to powder in liquid nitrogen. Replicates of 50 mg of powder were then stored at − 80 °C until DNA extraction.

### Soil invertebrates

Live Coleoptera from the families Staphilinidae and Carabidae (Invert_beetle) were collected from six sites between 1000 and 2500 m a.s.l. using sterile gloves (Moret et al. 2016), rinsed with a in 0.1 M Tris Saline Buffer to remove soil, pooled by site in sterile 50 ml polypropylene tubes, and stored at − 20 °C, following taxonomic identification using morphological keys (Freude, Harde, and Lohse 2012; Pesarini and Monzini 2010). Three frozen whole Coleoptera from each pool were placed in a sterile mortar, ground to powder with a sterile pestle in liquid nitrogen, then divided into six biological replicates (~ 30 mg of powder each) and stored at − 80 °C until DNA extraction. For technical replicates, the frozen Coleoptera powder from a single pool was split into 30 aliquots of ~ 10 mg each and stored at − 80 °C until DNA extraction (Table S1).

Live bacterivorous Nematoda (Invert_roundworm) were extracted from soil samples (5 cm in diameter, 5 cm deep) taken from 10 sites between 1000 and 2500 m a.s.l. following the Baermann funnel extraction protocol^60^. Using an optical microscope at 40X magnification, all nematodes from a single soil sample were pooled and pipetted into 0.1 M Tris Saline Buffer and stored at − 20 °C. Frozen Nematoda samples were thawed on ice, pooled in 2 ml DNA/DNase free tubes, mixed by gentle vortexing and divided into six biological or 30 technical replicates (Table S1). Aliquots were stored at -80 °C until DNA extraction.

### Soil samples

Soil was collected from the upper mineral horizon (Ah) just beneath the root system of the overlying plant community (Soil_horizonA), from one pasture at 1000 m a.s.l (Table S1). At three sites at least 2 m apart, a site sample with an area of 20 × 15 × 10 cm was taken for subsequent soil sampling at a depth of 12–20 cm; for each of the site samples, approximately 100 g of soil was collected from 5 to 10 such different digging points on the site sample and combined into one composite sample. For rhizosphere soil of the grass *Festuca* spp. (Soil_rhizosphere), 2–3 cespitoses (including roots) were dug out at the same three sites near the bulk soil (Soil_horizonA) sampling. Care was taken to ensure that individual cespitoses were isolated from others, that there were no foreign roots, and that there was enough soil sticking to the roots to protect the plant-root complex during transport. Soil and plant samples were transported at ambient temperature the same day to the laboratory and stored at 4 °C until further processing (up to two weeks).

To collect the rhizosphere soil samples, plant phyllosphere and roots were separated at the root collar using a sterile blade, and roots underwent a washing-centrifugation procedure as described in^37,61^. To allow the comparison of DNA extracts of rhizosphere soil and bulk soil (Horizon ‘A’ soil), the latter was sieved to < 1 mm and 100 mg of this fraction was subjected to the same washing-centrifugation protocol as the rhizosphere soils^37,61^. From each of the three sites, 6 aliquots (biological replicates) containing approximately 100 mg of bulk and rhizosphere soil were frozen at − 80 °C until DNA extraction. To generate technical replicates of bulk and rhizosphere soil respectively, aliquots of the soil suspensions from the three sites were pooled, mixed by vortexing, portioned in 30 tubes of equal amount and frozen at − 80 °C until DNA extraction.

### DNA extraction

For each sample type (Feces_cattle, Feces_hare, Invert_beetle, Invert_roundworm, Soil_horizonA, Soil_rhizosphere), DNA extractions were performed for three biological replicates (hereafter referred to as Pools A, B, C) and six technical replicates (Pool D) (Table S1). Three aliquots from pool D (D1mc, D2mc, D3mc) were extracted with a bacterial mock community before the initial lysis step, using the ZymoBIOMICS™ Spike-in Control I (High Microbial Load; EuroClone, Milan, Italy; hereafter MC; after^43^) and the other three aliquots (called D1, D2, D3) were extracted without the inclusion of MC. A single MC dose (20 μl, as defined by the manufacturers) includes 2 × 10^7^ cells, corresponding to 6.0 × 10^7^ (*I. halotolerans*) and 1.4 × 10^8^ (*A. halotolerans*) 16S rRNA gene copies). Therefore, the MC dose adopted for the different sample types were the following: 0.8 dose for Feces_cattle and Feces_hare; 0.33 dose for Invert_beetle; 0.12 dose for Invert_roundworm; and 1.0 dose for Soil_horizonA and Soil_rhizosphere.

DNA extractions were performed using the manufacturer’s protocols for the following kits: DNeasy® Blood & Tissue (QIAGEN; hereafter QBT), QIAamp® DNA Micro (QIAGEN; QMC), NucleoSpin® Soil (MACHEREY–NAGEL; MNS), DNeasy® PowerSoil® Pro (QIAGEN; QPS) and QIAamp® Fast DNA Stool Mini (QIAGEN; QST). These kits were selected because they are widely cited in microbial ecology studies; in addition, they are specifically recommended by manufacturers for one or more of the sample types considered in this study. For QBT, samples were subjected to the pre-treatment for gram-positive bacteria, followed by the protocol ‘Purification of Total DNA from Animal Tissues’. Extractions with QMC were performed using the protocol ‘Isolation of Genomic DNA from Tissues’. For MNS, lysis buffer SL1 and 50 μl of Enhancer SX were used as reported by^37^. For QST, we followed the protocol ‘Isolation of DNA from stool for pathogen detection’. For each kit, negative DNA extraction controls (lysis buffer only: no sample material) were included for contamination control. The purity and quantity of all DNA extracts were assessed by checking the UV/VIS spectra of each extract with a Spark® multimode microplate reader (Tecan, Switzerland). Estimated DNA concentration, and extracts purity expressed as 260/280 and 260/230 ratios were plotted as Log2 values boxplots generated using the R package ggplot2^62^; negative values were replaced with 0.001. Analysis of variance (ANOVA) was performed with the R function ‘aov’, and Tukey's test for post-hoc analysis was performed with the R package agricolae^63^.

### 16S rRNA gene amplification, library preparation and sequencing

Amplification reactions were performed in a volume of 30 μl, containing 9 ng of genomic DNA, 1X FastStart High Fidelity Reaction Buffer (Roche Applied Science), primers to a final concentration of 0.4 μM each and 1.5 U of FastStart High Fidelity Enzyme Blend (Roche Applied Science). The following primer pairs were adopted in all reactions: 515F-mod_ILL^8^ and 806R-mod_ILL^64^. All PCR reactions were performed on Veriti™ 96-Well Fast Thermal Cyclers (Applied Biosystems, USA). The experimental conditions for PCR amplification were as follows: 3 min at 95 °C, followed by 31 cycles of 45 s at 95 °C, 60 s at 55 °C, 90 s at 72 °C, and a single final extension step of 7 min at 72 °C. Non-template controls (amplification mix only: PCR-grade water instead of DNA template) were included for contamination control. Amplicons were visualized by high-resolution capillary electrophoresis using the QIAxcel Advanced System (QIAGEN). Quality and quantification of pooled amplicon libraries as well as high throughput sequencing by Illumina technology were performed at the Sequencing and Genotyping Platform, Fondazione Edmund Mach (FEM, San Michele all’Adige, Italy). Samples, DNA extraction negative controls and amplification blank controls were sequenced using Illumina MiSeq Standard Flow Cells targeting a minimum depth of 30,000 reads per sample. In total, we generated 284 amplicon libraries, which were named by specifying the DNA extraction kit (e.g. QBT, QMC, MNS, QPS, QST), followed by the sample identifier and the replicate number. For example, the library ‘MNS-BPD-1’ was generated using the extraction kit MNS from the Feces_cattle Pool D (BPD) and was identified as replicate 1 (see Table S1).

### Data analysis

Bioinformatic pre-processing of fastq reads was carried out with the software MICCA^65^ with default settings. Sequence Variants (SVs) were generated using UNOISE3^66^ implemented in MICCA. Taxonomy was assigned with the ‘classify.seqs’ command in MOTHUR^67^ and by using silva.nr_v138^68^ as the template and taxonomy reference files. To generate a phylogenetic tree, ASVs were aligned to the silva.nr_v138 sequences using Nearest Alignment Space Termination (NAST) approach implemented in MICCA. The generation of the phylogenetic tree and midpoint rooting were performed with default parameters in MICCA.

The following statistical analyses were performed with the R packages phyloseq^69^ and vegan^70^: ASVs matching the MC sequences (ACC: NR116607.1, NR117181.2) were extracted from the MC-control samples, verified by BLAST (https://blast.ncbi.nlm.nih.gov) and, when confirmed by the sequence alignment (number of ASVs: 17), removed from all datasets before performing subsequent steps. Following the removal of ASVs matching the MC, the dataset consisted of 26,996 ASVs. Separately for each DNA extraction kit, putative contaminant ASVs were detected using decontam v1.22. 0 with the prevalence method^71^. The number of putative contaminant ASVs was 140 for QBT (median prevalence: 2, range: 0–22), 13 for QMC (median prevalence: 2, range: 1–22), 398 for MNS (median prevalence: 2, range: 0–23), 34 for QPS (median prevalence: 2.5, range: 1–23) and 26 for QST (median prevalence: 1, range: 0–17). Considering that the detected median prevalence was so low that that decontam v1.22 was not effective in identifying cross contaminations^71^, and to avoid affecting comparisons across kits due to the filtering of sample taxa, we did not filter the potential contaminating ASVs from the dataset. To estimate alpha diversity indices (Richness, S; Shannon diversity, H and Inverse Simpson, D_2_), libraries were rarefied to 99% of the minimum sample depth in the dataset (21,675 reads per sample) using the R package phyloseq^69^. Log_2_ Euclidean distances of observed number of taxa (richness, S) across samples extracted with the same kit (referred as: *Within kit*), with different kits (*Between kits*), between biological replicates (*Between ind*) and across kits and biological replicates (*Between kits and ind*) were estimated with the R package agricolae^63^. Significant differences between estimates were tested using the Kruskal–Wallis test in the R package agricolae^63^ with a significance level of 0.05. Plots were generated with the R package ggplot2^62^.

To measure the effect of the explanatory variables, DNA concentration, 260/280 and 260/230 ratios on estimated alpha diversity (response variables: Richness, Shannon diversity) was estimated with Linear Mixed Model (LMM) using the R lmerTest package^72,73^.

To compare microbial communities generated from different DNA extraction kits, two reference microbial communities (hereafter, reference communities) were computationally generated for each sample type by merging the fastq files generated from the corresponding DNA extracts. Only technical replicates processed without MC were used to generate these reference communities. Fastq files of single libraries and reference communities were reanalyzed together with the software MICCA^65^ and R packages phyloseq^69^ and vegan^70^, as described above.

Beta diversity indices Bray–Curtis, weighted UniFrac and Jaccard were estimated with the R package phyloseq^69^ and vegan^70^. To compare beta-diversity estimates across samples, permutational MANOVA (permanova) statistical tests were performed by using the function ‘adonis’ with 999 permutations (R package vegan). Plots were generated with the R package ggplot2.

Differential abundance testing was carried out for each sample type using non-rarefied data with the package DEseq2^50^ in R^72^. To exclude biological variation and focus on the effect of DNA extraction methods on ASV differential abundances, only technical replicates (N = 3) were used. *p*-values were corrected for false discovery rate using the Benjamini–Hochberg correction implemented in DEseq2 (significance cutoff of FDR corrected *p*-values: 0.05). Taxonomic classification of enriched and deleted taxa and the corresponding barplots were performed with the R package phyloseq.

### Supplementary Information

Supplementary Figures.

Supplementary Tables.

## Data Availability

The raw sequencing data has been deposited at NCBI Sequence Read Archive (SRA) under the BioProject ID PRJNA949849.
