# Supplementary material for: Comparison of DNA extraction methods on different sample matrices within the same terrestrial ecosystem
Source: Sci Rep. 2024 Apr 15;14:8715. doi: 10.1038/s41598-024-59086-4 (PMC11018758; doi:10.1038/s41598-024-59086-4)
Supplement: Supplementary file 1 — Supplementary Figures. [file 41598_2024_59086_MOESM1_ESM.pdf]

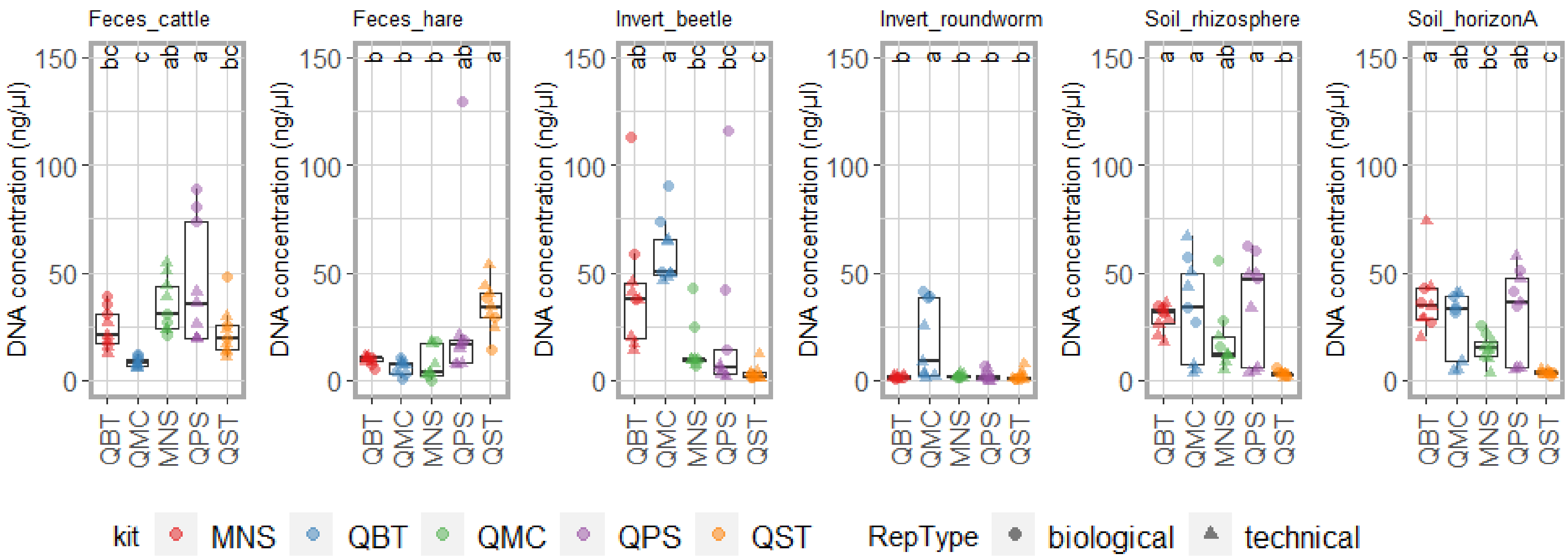

Figure S1. Concentration [ng/µl] of DNA extracts in each sample. DNA concentration was estimated by UV/VIS analysis. Samples are colored according to the DNA extraction method as indicated in the plot legend (kit). Biological and technical replicates are shown with different shape formats as indicated in the plot legend (RepType). Letters in the above area of the graph indicate significant differences in DNA concentration across DNA method (Kruskal-Wallis test, $p<0.05$).

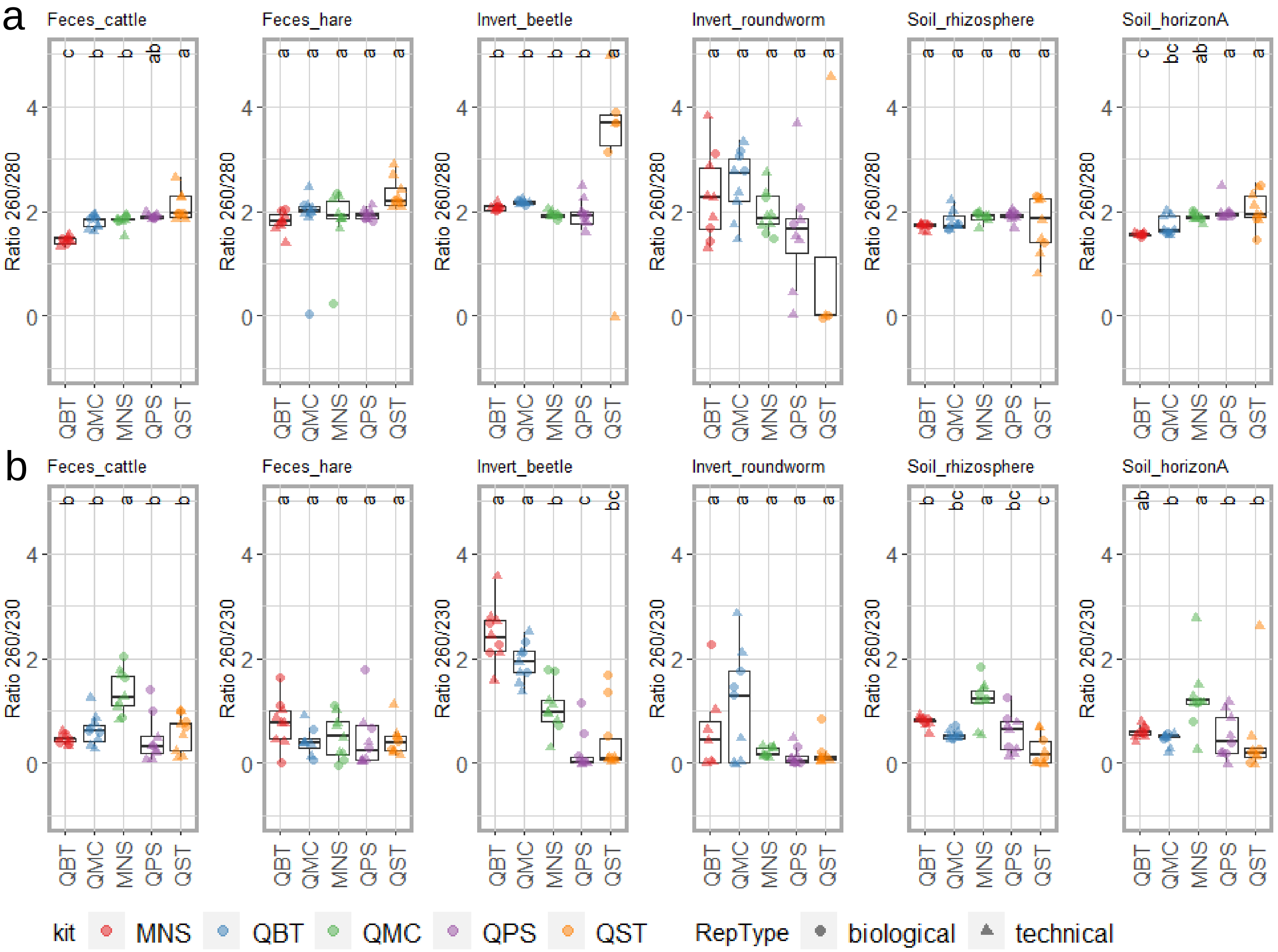

Figure S2. Quality of DNA extracts in each sample estimated by UV/VIS analysis expressed as ratio between 260 nm and 280 nm absorbance values (panel a) as well as 260 nm and 230 nm absorbance values (panel b). Samples are colored according to the DNA extraction method as indicated in the plot legend. Biological and technical replicates are shown with different shape formats as indicated in the plot legend (RepType). Letters in the above area of the graph indicate the grouping resulting from Kruskal-Wallis test on diversity estimates ($p<0.05$).

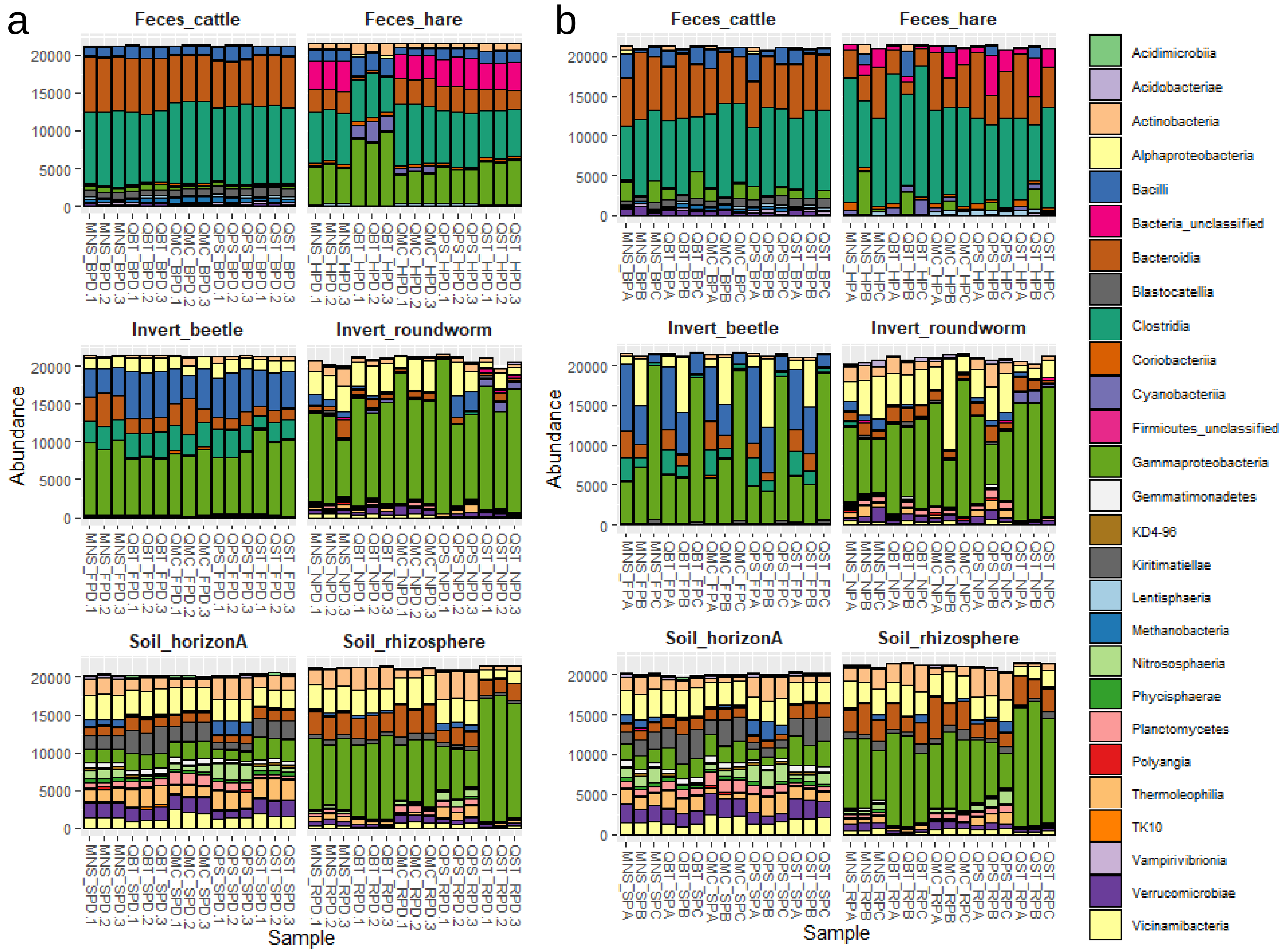

Figure S3. Taxonomic classification by class of microbiota detected in technical (A) and biological (B) replicates of each sample type processed with different DNA extraction methods (plot legend). Following the rarefaction to 21675 reads/sample, only taxa with a minimum abundance of 100 reads (~0.46% of sample reads) in at least 15% of the samples (~13.5 samples out of 90) were plotted. Samples were named by specifying the DNA extraction kit (e.g. QBT, QMC, MNS, QPS, QST), followed by the sample identifier and the replicate number. For example, the library QST-RPD.3 was generated using the extraction kit QST from the rhizosphere soil Pool D (RPD) and was identified as replicate 3.

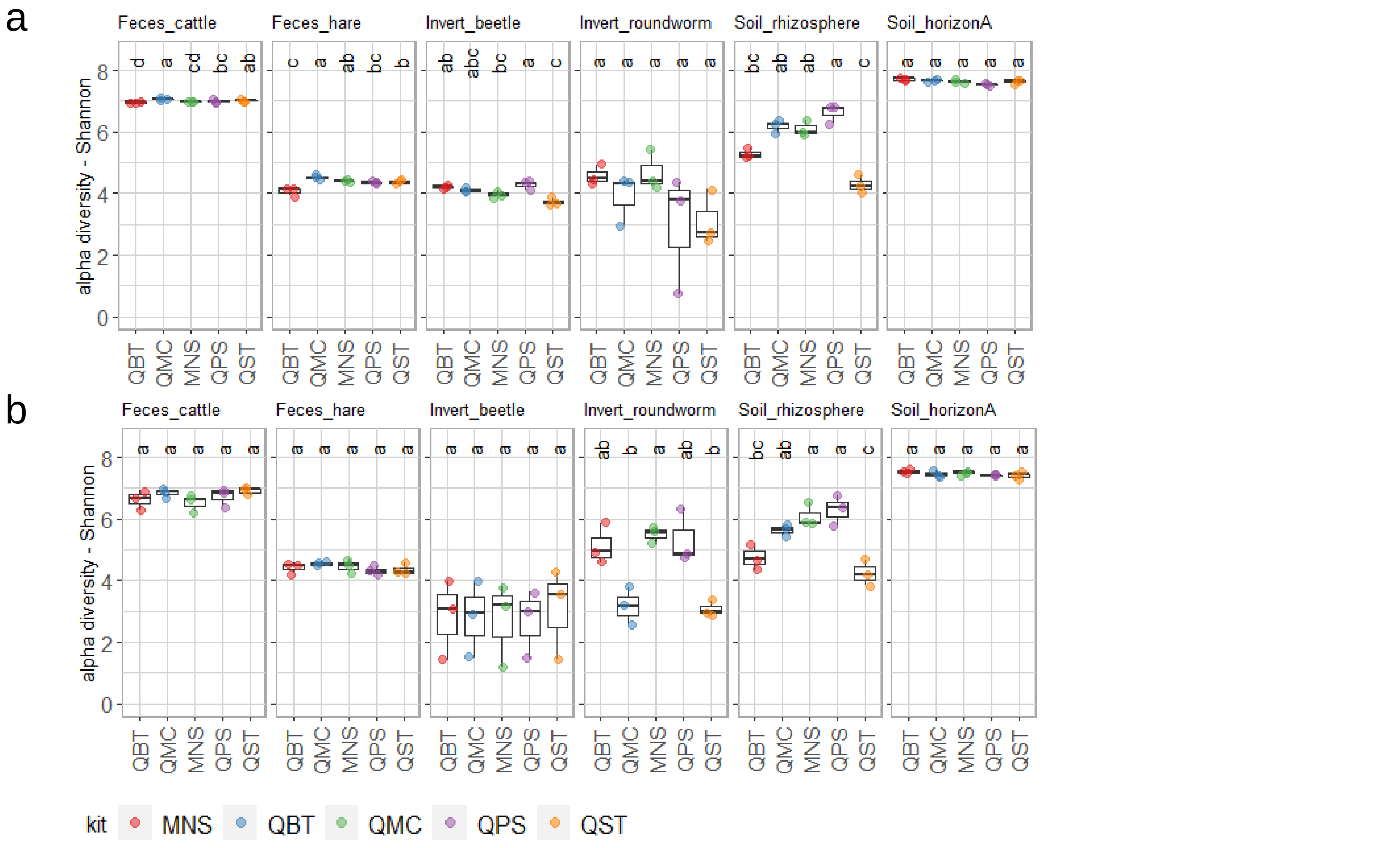

Figure S4. Shannon diversity estimates across sample types and kits. Panel a: technical replicates; Panel b: biological replicates. Samples are clustered by sample type and colored according to the DNA extraction method as indicated in the plot legend (kit). Letters in the above area of graphs indicate the grouping resulting from Kruskal-Wallis tests on Shannon diversity estimates between samples extracted with different kits. QBT: DNeasy® Blood & Tissue (QIAGEN); QMC: QIAamp® DNA Micro (QIAGEN); MNS: NucleoSpin® Soil (MACHEREY-NAGEL); QPS: DNeasy® PowerSoil® Pro (QIAGEN); QST: QIAamp® Fast DNA Stool Mini (QIAGEN).

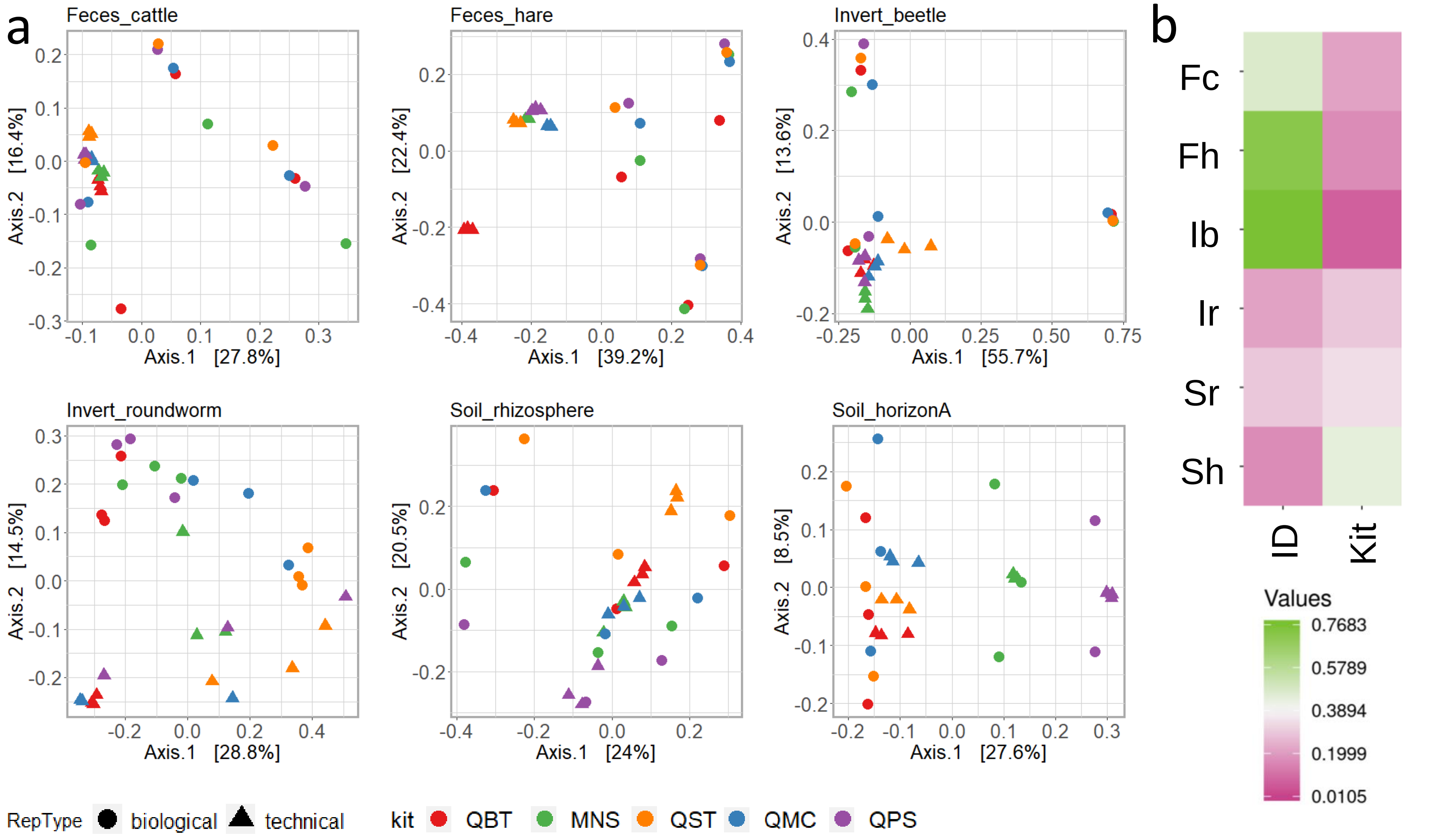

Figure S5. Panel a: Beta diversity estimates (Bray-Curtis) on samples processed with different DNA extraction protocols. Technical and biological replicates are shown. Each plot refers to a different sample type. Technical and biological replicates are marked with different dot shape as reported in the figure legend. Samples are colored according to the DNA extraction method as indicated in the plot legend (kit). Panel b: Variance in diversity estimates explained in PERMANOVA analyses (R_2) by the following variables: individual (ID) and DNA extraction method (kit). Fc: Feces_cattle; Fh: Feces_hare; Ib: Invert_beetle; Ir: Invert_roundworm; Sr: Soil_rhizosphere; Sh: Soil_horizonA

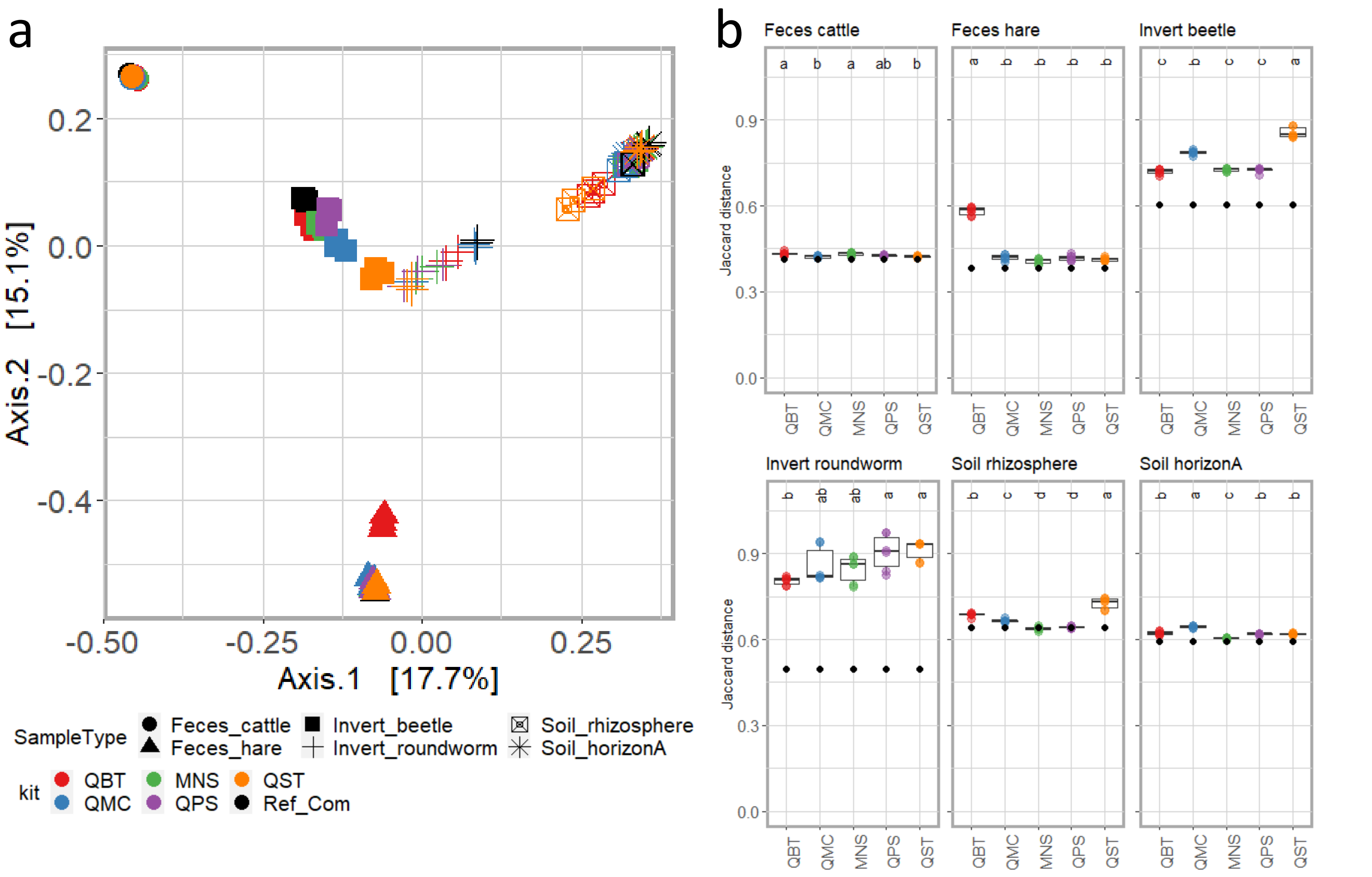

Figure S6. Beta diversity estimates across trans-samples and respective samples. Panel a: PCoA was generated by using and Jaccard distance. The percentage of the total variance explained by the axis is reported in the figure. Sample types are marked with different shape formats as indicated in the plot legends (SampleType). Samples are colored according to the DNA extraction method as indicated in the plot legend (kit). Panel b: Distance across trans-samples (black dots) and the corresponding samples. Samples are colored according to the DNA extraction kits as indicated in the plot legend (kit). Letters in the above area of graphs indicate the grouping resulting from Kruskal-Wallis tests on Jaccard distances between trans-samples and the corresponding samples extracted with different kits.
